# Enrichment of ^150^Nd for neutrinoless double-beta decay detection

**DOI:** 10.1038/s41598-022-15597-6

**Published:** 2022-07-06

**Authors:** M. V. Suryanarayana

**Affiliations:** Bhabha Atomic Research Centre, Visakhapatnam, Andhra Pradesh India

**Keywords:** Atomic and molecular collision processes, Atomic and molecular interactions with photons, Quantum optics, Electronic structure of atoms and molecules

## Abstract

A 562 nm–627 nm–597 nm three-step resonant photoionization scheme has been studied using the density matrix formalism for the enrichment of ^150^Nd in weighable quantities for the neutrinoless double-beta decay detection. The effect of bandwidth of the excitation laser and charge exchange collisions, on the production rates and degree of enrichment has also been studied. Optimum conditions for the efficient enrichment of ^150^Nd isotope have been derived. It has been shown that it might be possible to produce 50 kg of 66% enriched ^150^Nd isotope in about five months (16 h/day) using the conditions derived through this investigation. This enables to reach the 0νββ decay half-life limit of ≈ 1.8 × 10^25^ year for the ^150^Nd isotope.

## Introduction

Neutrinos are fundamental particles of the standard model of particle physics and are the only fermions that have no charge. According to the standard model, neutrinos (also known as Dirac fermions) have distinct anti-neutrinos. It has also been predicted that the neutrinos can also be their own anti-neutrinos which are called Majorana neutrinos^1^. The distinction between the two types of neutrinos could not be established experimentally. Neutrinoless double-beta decay (denoted as 0νββ decay) of an atomic nucleus, which is a lepton number violating process, confirms the presence of Majorana neutrinos. However, the detection of neutrinoless double beta decay process is utmost challenging. The current best limit for the 0νββ decay T_1/2_ > 1.07 × 10^26^ year, was obtained by the KamLAND-Zen experiment^2^ with ^136^Xe.

The search for the 0νββ decay consists of detection of the two emitted electrons. Due to the negligible recoil of the nucleus, the sum of energies of the two electrons corresponds to the Q-value of the process. Therefore, the signature of the 0νββ decay is a mono-energetic peak centred at Q_0νββ_. The sensitivity of the detection of 0νββ decay depends on the ratio of signal events to the background events, which can be expressed as^3^

1 $${S}^{0\nu \beta \beta }=\text{ln}2\cdot \left(\frac{{\text{N}}_{\text{A}}}{{\text{m}}_{\text{A}}}\right)\cdot \left(\frac{\varepsilon \cdot \text{f}}{{\text{n}}_{\upsigma }}\right)\cdot \sqrt{\frac{\text{M} \cdot \text{T}}{\text{B} \cdot {\Delta E}}}$$

where, N_A_ is the Avogadro number, m_A_ is the molar mass of the 0νββ emitter, ε is the detection efficiency, f is the fractional abundance of the isotope in the detector, n_σ_ is the confidence level of the detector, M is the mass of the detector, T is the time period of the measurement, ∆E is the region of interest (ROI) width.

Assuming the detectors being the same, for the same amount of 0νββ emitter and measured for the same time period; the sensitivity of the detection is proportional the ratio of the fractional abundance and the molar mass of the 0νββ emitter.

2 $${S}^{0\nu \beta \beta }\propto \left(\frac{\text{f}}{{\text{m}}_{\text{A}}}\right)$$

A total of nine isotopes, namely, ^48^Ca, ^76^Ge, ^82^Se, ^96^Zr, ^100^Mo, ^116^Cd, ^130^Te, ^136^Xe and ^150^Nd are under investigation for the 0νββ decay (Table 1). Among these isotopes, the isotopes ^48^Ca and ^150^Nd are particularly of interest due to the high Q_0νββ_ (> 3.3 MeV) value which enables detection of 0νββ decay with low background.

**Table 1:** A list of isotopes of interest and the Q-values for the neutrinoless double beta decay.

Isotope	Natural Abundance (%)	Q-value
^48^Ca	0.187	4.2737
^76^Ge	7.8	2.0391
^82^Se	9.2	2.9551
^96^Zr	2.8	3.350
^100^Mo	9.6	3.0350
^116^Cd	7.5	2.805
^130^Te	34.5	2.5303
^136^Xe	8.9	2.4578
^150^Nd	5.6	3.3673

From the Eq. (2), an isotope with higher natural abundance and smaller mass provides higher sensitivity for the detection 0νββ decay. Ideally, to minimize the costs, the 0νββ emitter should be available in the earth crust in large quantities and preferentially in highest isotopic purity. Unfortunately such an isotope is not available naturally.

Though the isotope ^130^Te has the highest natural abundance of 34.5%, the low Q-value (2.5303 MeV) of the process makes it difficult to detect the 0νββ decay above background events. On the other hand, despite having the high Q-value, the low natural abundance of ^48^Ca (0.187%) and ^150^Nd (5.6%) makes these isotopes rather unattractive. Arnold et al^4^ have made measurements for 5.25 years of 0νββ decay of ^150^Nd to the 0_1_^+^ and 2_1_^+^ nuclear states of ^150^Sm using 36.6 g of 91% enriched ^150^Nd. Based on the number of events observed within the 2–2.8 MeV energy range under the 0νββ peak of the two-electron energy spectrum, the half-life limits for the 0νββ decay to the two states were determined to be T_1/2_ (0_1_^+^) ≈ 1.36 × 10^22^ year and T_1/2_ (2_1_^+^) ≈ 1.26 × 10^22^ year respectively at a 90% confidence level. However, these significantly fall shorter than the theoretically predicted^5,6^ values of 2.6 × 10^23^ year and 7.2 × 10^24^ year. Theoretically predicted half-life can only be achieved using > 50 kg of enriched ^150^Nd. Atomic Vapor Laser Isotope Separation (AVLIS) seems to be the only viable method for the enrichment of this isotope at the required quantities.

Calcium, which is the fifth most abundant element in the earth crust (elemental abundance of ~ 4.1%) can be a natural choice for the enrichment in ^48^Ca isotope. Nevertheless, enrichment of ^48^Ca isotope through AVLIS is considerably more complex than ^150^Nd for the following reasons.

1. The wavelengths of the photoionization scheme are not accessible by high efficiency-high repetition rate tunable laser systems^7^
2. Small isotope shifts for the Ca transitions^8,9^
3. Small natural abundance (0.187%) of ^48^Ca isotope.

Nonetheless, recently, deflection method has been reported^10^ as a proof-of-principle experiment for the separation of ^48^Ca isotope. On the other hand, Nd having ground state configuration of [Xe] 4f^4^ 6s^2^ has a rich electronic spectrum. As a result, it is possible to find suitable photoionization schemes accessible by high repetition rate tunable laser systems. Further, the isotope shifts of the Nd transitions are significantly higher than the case of Ca transitions^11^.

Russian AVLIS group^12–14^ has extensively worked towards the enrichment of ^150^Nd isotope. They have achieved 60% enrichment of ^150^Nd with a production rate of 40 mg/h and 65% of enrichment with a production rate of 25 mg/h. In view of the recent favourable results^4^ on 0νββ decay, the enrichment of ^150^Nd has been investigated for the improvement in terms of production rates and degree of enrichment. Density matrix formalism has been adopted for the investigation.

## AVLIS of Nd

Neodymium is a lanthanide element, having a ground state configuration of [Xe] 4f^4^ 6s^2^ and an ionization potential of 44,562 cm^−1^. It has seven natural isotopes, namely ^142^Nd (27.2%), ^143^Nd (12.2%), ^144^Nd (23.8%), ^145^Nd (8.3%), ^146^Nd (17.2%), ^148^Nd (5.7%) and ^150^Nd (5.6%). At the evaporation temperature (1500 °C) of Neodymium, the low lying meta-stable states 4f^4^6s^2^ ^5^I_4_, 4f^4^6s^2^ ^5^I_5_ and 4f^4^6s^2^ ^5^I_6_ will have a population of 54.4%, 26.6% and 11.5% respectively. Therefore, the photoionization pathway of Nd shall originate from the 4f^4^6s^2^ ^5^I_4_ ground state.

Nd can be photoionized through a three step resonant process involving photons in the yellow–red colour of the electromagnetic spectrum. Surprisingly, in comparison to other lanthanide elements, very little experimental work has been reported^15^ on the multi-step photoionization of Nd. Eight excitation transitions^16^ are available between the wavelength range of 562–734 nm originating from the ground state of Nd. Babichev et al.^12^ have carried out investigations on the three-step photoionization schemes originating from the 596.6 nm, 628.8 nm, 645.5 nm first excitation transitions. In one of the recent works, D’yachkov et al.^17^ have studied 3 three-step photoionization pathways wherein ionization is carried out through 597.24 nm transition in all cases. Among them the following photoionization scheme is attractive for the reasons mentioned below.

## Photoionization scheme

$$ \begin{aligned} & 4f^{4} 6s^{2} {}^{5}I_{4} \left( {0.0\;{\text{cm}}^{ - 1} } \right) \mathop{\longrightarrow} \limits^{{562.2086\;{\text{nm}}}} 4f^{3} 5d6s^{2} {}^{5}H_{3}^{o} \left( {17786.992\;{\text{cm}}^{ - 1} } \right)\mathop{\longrightarrow} \limits^{{627.38\;{\text{nm}}}} J \\ & \quad = 2\;or\;3\left( {33726\;{\text{cm}}^{ - 1} } \right)\mathop{\longrightarrow} \limits^{{597.21\;{\text{nm}}}} 50474\;{\text{cm}}^{ - 1} \;Autoionization\;State \to Nd^{ + } \\ \end{aligned} $$

1. The isotope shift (Table 2) between the adjacent isotopes ^148^Nd and ^150^Nd for the 562.2086 nm first excitation transition is large (1131 MHz).
2. The oscillator strength for the excitation transitions in the photoionization pathway is adequate^18,19^.
3. The wavelength of the autoionization transition lies within the high efficiency region of the dye lasers pumped by high-repetition rate Copper Vapor Laser (CVL) or Nd:YAG laser systems.
4. The cross-section^17^ for the autoionization transition is 5 × 10^–16^ cm^2^ which is quite adequate for the ionization of the Nd isotopes.

**Table 2:** Isotope shifts^17^ of the Nd isotopes for the 562.2086 nm first excitation transition with reference to the ^150^Nd isotope.

Isotope Shift with reference to ^150^Nd (MHz)					
^142^Nd	^143^Nd	^144^Nd	^145^Nd	^146^Nd	^148^Nd
− 3048.0	− 3060.0^a^	− 2448.0	− 2300.0^a^	− 1833.0	− 1131.0

^a^Isotope shifts of odd ^143^Nd and ^145^Nd isotopes has been calculated using the King’s plot analysis.

Nonetheless, the second excitation transition viz. the 627.38 nm transition has not been investigated and the isotopes shifts of Nd transitions have not been reported so far. Normally, the isotope shift of the upper level transitions is much smaller than the transitions from the lower levels; therefore, it can be ignored. Stockett et al.^19^ have reported transition probability of 13.1 ± 2.0 MHz for the 562.2068 nm transition corresponding to a lifetime of 76.3 ns. Based on the transitions from the upper levels, the transition probability^16^ of the 627.38 nm transition has been approximated to 20 MHz. Further, the contribution of the odd ^143^Nd and ^145^Nd isotopes in the enrichment of ^150^Nd has been ignored as they lie far away from the resonance of the ^150^Nd target isotope.

## Theoretical basis

**Figure 1:**
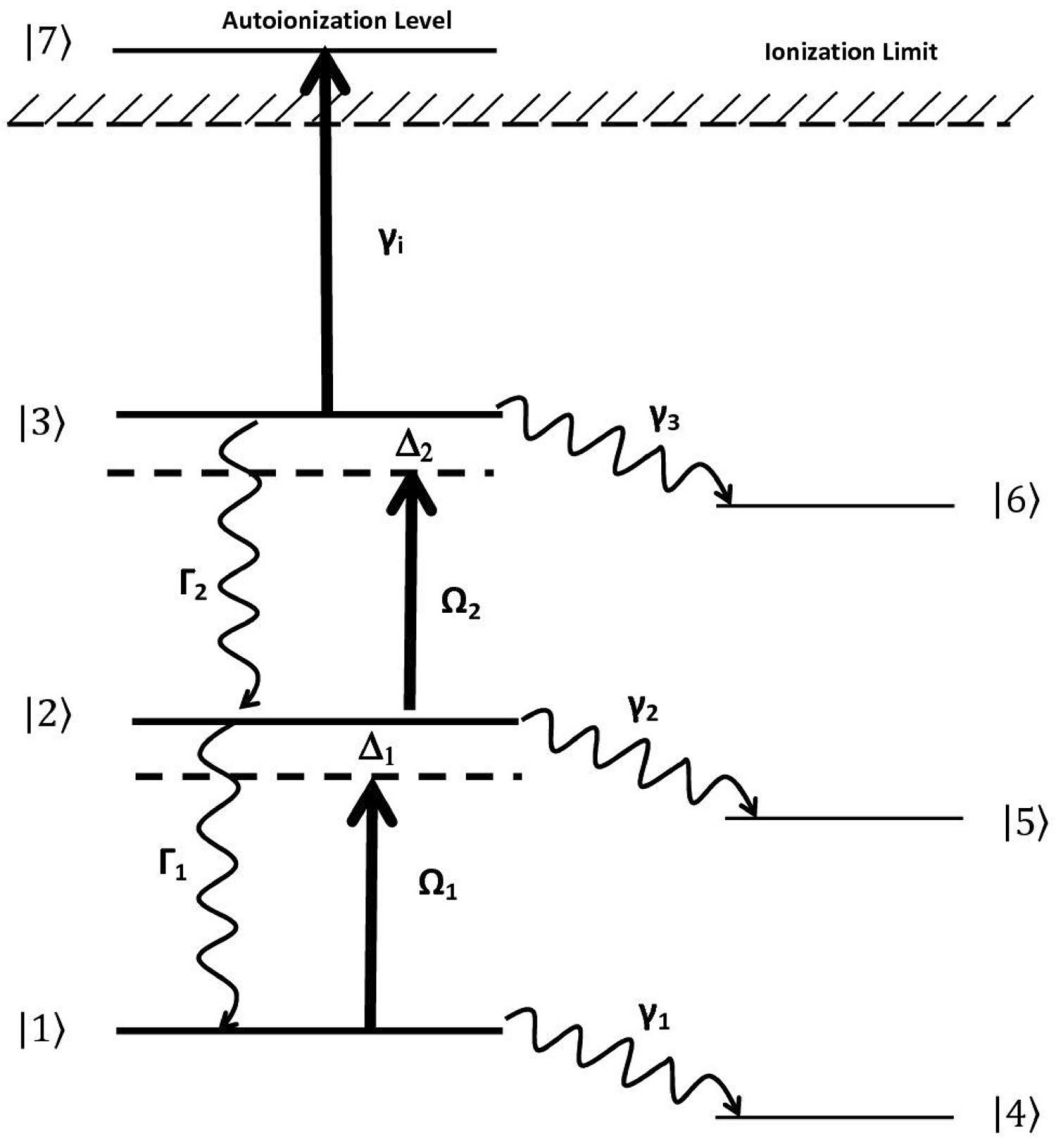
Schematic representation of the resonant three step photionization of Neodymium (not to scale).

Density matrix formalism accurately describes laser-atom interactions in the multi-step laser photoionization process^20^. A generalized three-step photoionization scheme is shown in Fig. 1. The ground, first and second excited fine-structure states are labelled as $\left|1\rangle \right.$, $\left|2\rangle \right.$ and $\left|3\rangle \right.$. The atoms initially present in the $\left|1\rangle \right.$ ground fine-structure level are excited by the first excitation laser having energy $\hslash {\omega }_{1}$ to the first excited level $\left|2\rangle \right.$. The atoms from the first excited level are further excited to the second excited level $\left|3\rangle \right.$ using second excitation laser having energy $\hslash {\omega }_{2}$, which are further incoherently transferred to the autoionization level by absorbing photons from a third laser and are eventually ionized. The atoms in a resonant upper level may decay to the resonant lower level $\left|I\rangle \right.$ at a rate denoted as Γ_I_. The atoms in resonant level $\left|I\rangle \right.$ also decay to trapped level at a rate denoted as γ_I_ and lost from the excitation process. The population dynamics in a three-step ladder excitation for an even isotope are described by the coupled density matrix equations given below.

3 $${\dot{\uprho }}_{11}=-i \cdot {\Omega }_{1}^{*} \cdot {\uprho }_{12}+i \cdot {\Omega }_{1} \cdot {\uprho }_{21}+2 \cdot {\Gamma }_{1} \cdot {\uprho }_{22}-2 \cdot {\gamma }_{1} \cdot {\uprho }_{11}$$

4 $${\dot{\uprho }}_{22}=-i \cdot {\Omega }_{1} \cdot {\uprho }_{21}-i \cdot {\Omega }_{2}^{*} ) \cdot {\uprho }_{23}+i \cdot {\Omega }_{1}^{*} \cdot {\uprho }_{12}+i \cdot {\Omega }_{2} \cdot {\uprho }_{32}+2 \cdot {\Gamma }_{2} \cdot {\uprho }_{33}-2 \cdot {(\Gamma }_{1}+{\gamma }_{2})  \cdot {\uprho }_{22}$$

5 $${\dot{\uprho }}_{33}=-i \cdot {\Omega }_{2} \cdot {\uprho }_{32}+i \cdot {\Omega }_{2}^{*} \cdot {\uprho }_{23}-2 \cdot \left({{\Gamma }_{2}+\gamma }_{3}+{\gamma }_{i}\right) \cdot {\uprho }_{33}$$

6 $${\dot{\uprho }}_{12}=i \cdot {\Omega }_{1} \cdot ({\uprho }_{22}-{\uprho }_{11})-i \cdot {\Omega }_{2 }^{*} \cdot {\uprho }_{13}-\left[i {\Delta }_{1}+{\Gamma }_{1}+{\Gamma }_{2}+{\gamma }_{1}+{\gamma }_{2}+2 \cdot {\gamma }_{L1}\frac{{\beta }_{1}^{2}}{{\Delta }_{1}^{2}+{\beta }_{1}^{2}}\right] \cdot {\uprho }_{12}$$

7 $${\dot{\uprho }}_{21}=-i \cdot {\Omega }_{1}^{*} \cdot ({\uprho }_{22}-{\uprho }_{11)}+i \cdot {\Omega }_{2} \cdot {\uprho }_{31}-\left[-i {\Delta }_{1}+{\Gamma }_{1}+{\Gamma }_{2}+{\gamma }_{2}+2 \cdot {\gamma }_{L1}\frac{{\beta }_{1}^{2}}{{\Delta }_{1}^{2}+{\beta }_{1}^{2}}\right] \cdot {\uprho }_{21}$$

8 $${\dot{\uprho }}_{23}=i \cdot {\Omega }_{2} \cdot {({\uprho }_{33}-\uprho }_{22})+i \cdot {\Omega }_{1}^{*} \cdot {\uprho }_{13}-\left[i {\Delta }_{2}+{\Gamma }_{1}+{\Gamma }_{2}+{\gamma }_{2}+{\gamma }_{3}+{\gamma }_{i}+2 \cdot {\gamma }_{L2}\frac{{\beta }_{2}^{2}}{{\Delta }_{2}^{2}+{\beta }_{2}^{2}}\right] \cdot {\uprho }_{23}$$

9 $${\dot{\uprho }}_{32}=-i \cdot {\Omega }_{2}^{*} \cdot {({\uprho }_{33}-\uprho }_{22})-i \cdot {\Omega }_{1} \cdot {\uprho }_{31}-\left[-i {\Delta }_{2}+{\Gamma }_{1}+{\Gamma }_{2}+{\gamma }_{2}+{\gamma }_{3}+{\gamma }_{i}-2 \cdot {\gamma }_{L2}\frac{{\beta }_{2}^{2}}{{\Delta }_{2}^{2}+{\beta }_{2}^{2}}\right] \cdot {\uprho }_{32}$$

10 $${\dot{\uprho }}_{13}=i \cdot {\Omega }_{1} \cdot {\uprho }_{23}-i \cdot {\Omega }_{2} \cdot {\uprho }_{12}-\left[i \cdot \left({\Delta }_{1}+{\Delta }_{2}\right)+{\Gamma }_{1}+{\Gamma }_{2}+{\gamma }_{1}+{\gamma }_{3}+2 \cdot {\gamma }_{L1}\frac{{\beta }_{1}^{2}}{{\Delta }_{1}^{2}+{\beta }_{1}^{2}}+2 \cdot {\gamma }_{L2}\frac{{\beta }_{2}^{2}}{{\Delta }_{2}^{2}+{\beta }_{2}^{2}}+{\gamma }_{i}\right]{\uprho }_{13}$$

11 $${\dot{\uprho }}_{31}=-i \cdot {\Omega }_{1}^{*} \cdot {\uprho }_{32}+i \cdot  \cdot {\Omega }_{2}^{*} \cdot {\uprho }_{21}-\left[-i \left({\Delta }_{1}+{\Delta }_{2}\right)+{\Gamma }_{1}+{\Gamma }_{2}+{\gamma }_{1}+{\gamma }_{3}+2 \cdot {\gamma }_{L1}\frac{{\beta }_{1}^{2}}{{\Delta }_{1}^{2}+{\beta }_{1}^{2}}+2 \cdot {\gamma }_{L2}\frac{{\beta }_{2}^{2}}{{\Delta }_{2}^{2}+{\beta }_{2}^{2}}+{\gamma }_{i}\right] \cdot {\uprho }_{31}$$

12 $$\text{Trapped State }{\dot{\uprho }}_{44}=+2 \cdot {\gamma }_{1} \cdot {\uprho }_{11}$$

13 $$\text{Trapped State }{\dot{\uprho }}_{55}=+2 \cdot {\gamma }_{2} \cdot {\uprho }_{22}$$

14 $${\text{Trapped State }}{{\dot{\uprho }}_{66}=+2 \cdot {\gamma }_{3} \cdot {\uprho }_{33}}$$

15 $$\text{Ion State }{\dot{\uprho }}_{77}=+2 \cdot {\gamma }_{i} \cdot {\uprho }_{33}$$

where the density matrix element ${\uprho }_{\text{MN}}$ represents the coherence between the states $\left|M\rangle \right.$ and $\left|N\rangle \right.$ when $M\ne N$ and represents the level population when $M=N$. ∆ is the detuning of the laser frequency from the atomic resonance frequency. Ionization is considered as an incoherent process, which is induced by ionizing laser at a rate ${\gamma }_{i}= \sigma \phi$, σ is the photoionization cross-section and ϕ is the flux of the ionization laser.

The effect of the laser bandwidth and its lineshape are included in the terms^21^.

16 $$2 \cdot {\gamma }_{i}\frac{{\beta }_{i}^{2}}{{\Delta }_{i}^{2}+{\beta }_{i}^{2}},\text{i}=1, 2$$

According to the phase diffusion model, the laser spectrum is Lorentzian near the centre with full width at half maximum (FWHM) γ_i_ and has a cut-off around β_i_. For the detunings ∆_i_$\ll$β_i,_ these terms reduce γ_i_; while ∆_i_$\gg$β_i,_ the laser appears to be monochromatic.

In principle, the atomic ensemble will have a distribution of velocities. The flux distribution of atomic ensemble is given by^21^

17 $$\phi \left(v\right)=2\left(\frac{{v}^{3}}{{\alpha }^{4}}\right) \cdot {e}^{-\left(\frac{{v}^{2}}{{\alpha }^{2}}\right)}dv$$

where, α is the most probable velocity and for the present numerical calculations the integration is carried out up to 4α, at which the relative flux has dropped to the value of ~ 10^–7^ of the maximum. For a typical atomization temperature of 1500 °C for Nd, the most probable velocity of ^150^Nd is ~ 443 m/s. For the entire calculations in this work, the pulse width of the laser is considered to be having a Gaussian pulse-width of 18 ns, no delay between the pulses and all the lasers are co-propagating unless stated otherwise. Initially, the population of the ground state is set to one. The coupled density matrix equations are then integrated using the standard numerical integration method for the set conditions for the entire duration of the laser–atom interaction. At the end of the laser interaction, the population of the ion state corresponds to the ionization efficiency of the photoionization process. For the inclusion of Doppler broadening of the atomic ensemble in the calculations, divergence angle is segmented into a minimum of 30 angular groups; and each angular group is segmented into a minimum of 30 velocity groups.

## Results and discussion

**Figure 2:**
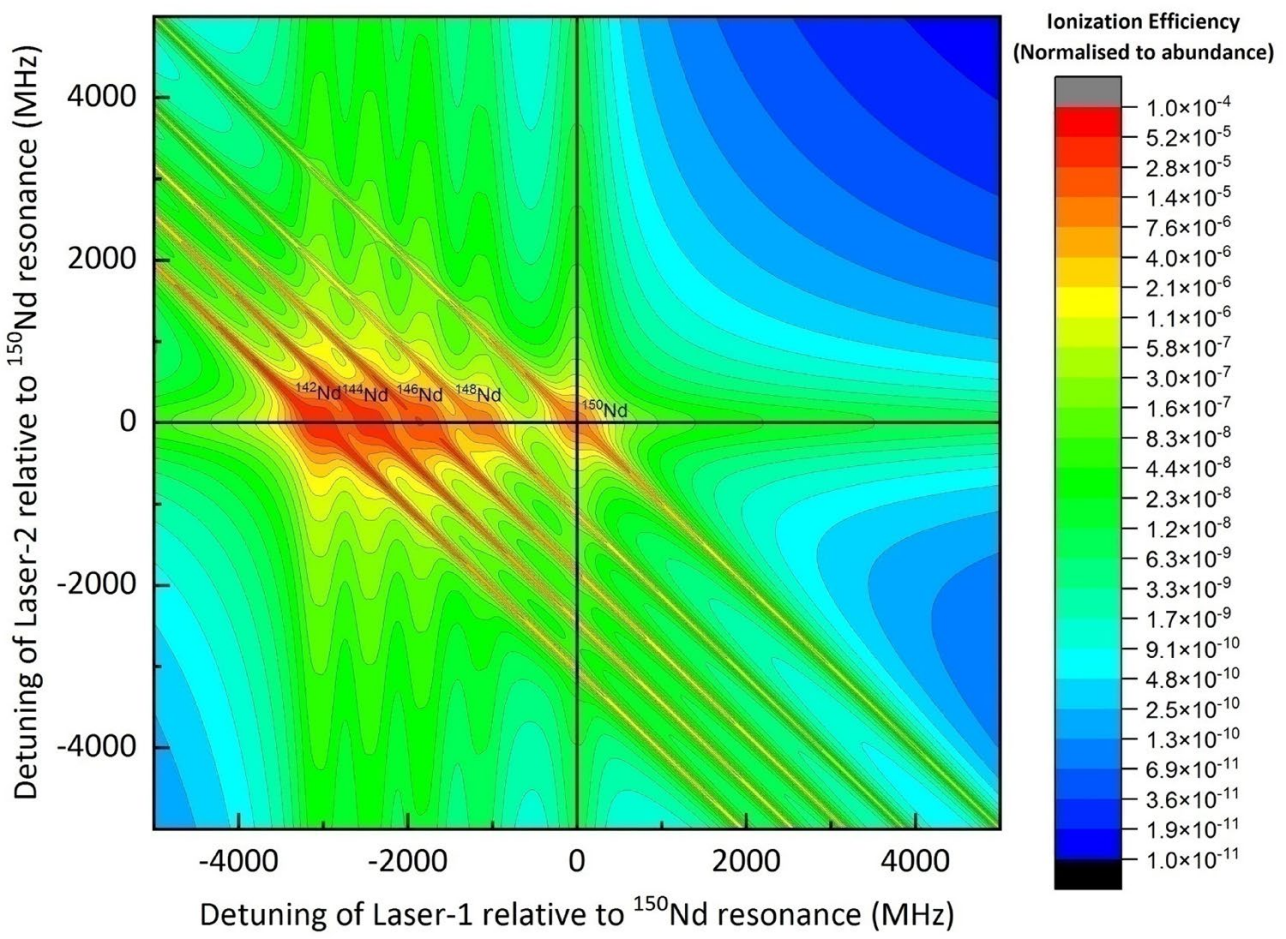
Two-dimensional contour plot of ionization efficiencies of Nd (frequencies relative to the resonance of ^150^Nd) for the Doppler-free condition. Bandwidth of all the excitation lasers is set to 100 MHz, and the peak power densities of all excitation lasers are set to 5 W/cm^2^.

Ionization efficiency of all the constituent isotopes has been calculated by varying the frequency of both the excitation lasers for all the isotopes of Nd by setting the velocity and angular distributions to zero (i.e., under Doppler free conditions). The ionization efficiency of each isotope has been normalized to the abundance of the constituent isotopes. The resultant contour has been shown in Fig. 2. From Fig. 2, it can be seen that the resonance frequency positions of all the constituent isotopes agreed well with the respective isotope shift values (Table 2). The vertical ridges observed for each isotope correspond to the excitation spectrum of the second step while the horizontal ridges correspond to the excitation spectrum of the first step. The diagonal ridges correspond to the coherent two-photon ionization. The diagonal ridges arise due to the coherent two-photon ionization when the sum total energy of the two photons is equal to the energy of the upper 33726 cm^−1^ level. A detailed discussion on the lineshapes in the two-step-photoionization process can be found elsewhere^7,22^.

In a laser isotope separation process, degree of enrichment can be calculated from the ionization efficiency of the constituent isotopes using the following expression

18 $$\text{Degree of enrichment }\left(\text{\%}\right)\text{ of }{}^{150}\text{Nd }=\left\{\frac{{\upeta }_{{150}_{\text{Nd}}}{*\text{A}}_{{150}_{\text{Nd}}}}{\sum_{x}^{all\;isotopes}{\upeta }_{{\text{x}}_{\text{Nd}}}{*\text{A}}_{{\text{x}}_{\text{Nd}}}}\right\}*100$$

where A is the initial fractional abundance of the isotope and η is the ionization efficiency.

When the lasers are tuned to the resonance of ^150^Nd isotope, under the conditions described in Fig. 2, ionization efficiency (normalized to the natural abundance) of ^150^Nd ≈ 10^–5^ can be achieved. The low ionization efficiency can be attributed to the low peak power density (5 W/cm^2^) of the excitation and ionization lasers. Such low ionization efficiency is grossly inadequate for an efficient laser isotope separation process. In order to increase the ionization efficiency, it is essential to increase the powers of the excitation and ionization lasers. This increase causes power broadening of the resonance lines. Further, in a realistic laser isotope separation process, atomic ensemble consists of angular and velocity distributions which result in Doppler broadening. As a result of both power broadening and Doppler broadening, the resonance lines of the isotopes are broadened resulting in the loss of degree of enrichment. Therefore, high degree of enrichment and high ionization efficiency demand mutually exclusive conditions. Thus one needs to find optimum conditions wherein desired degree of enrichment can be obtained without significant sacrifice in the ionization efficiency.

**Figure 3:**
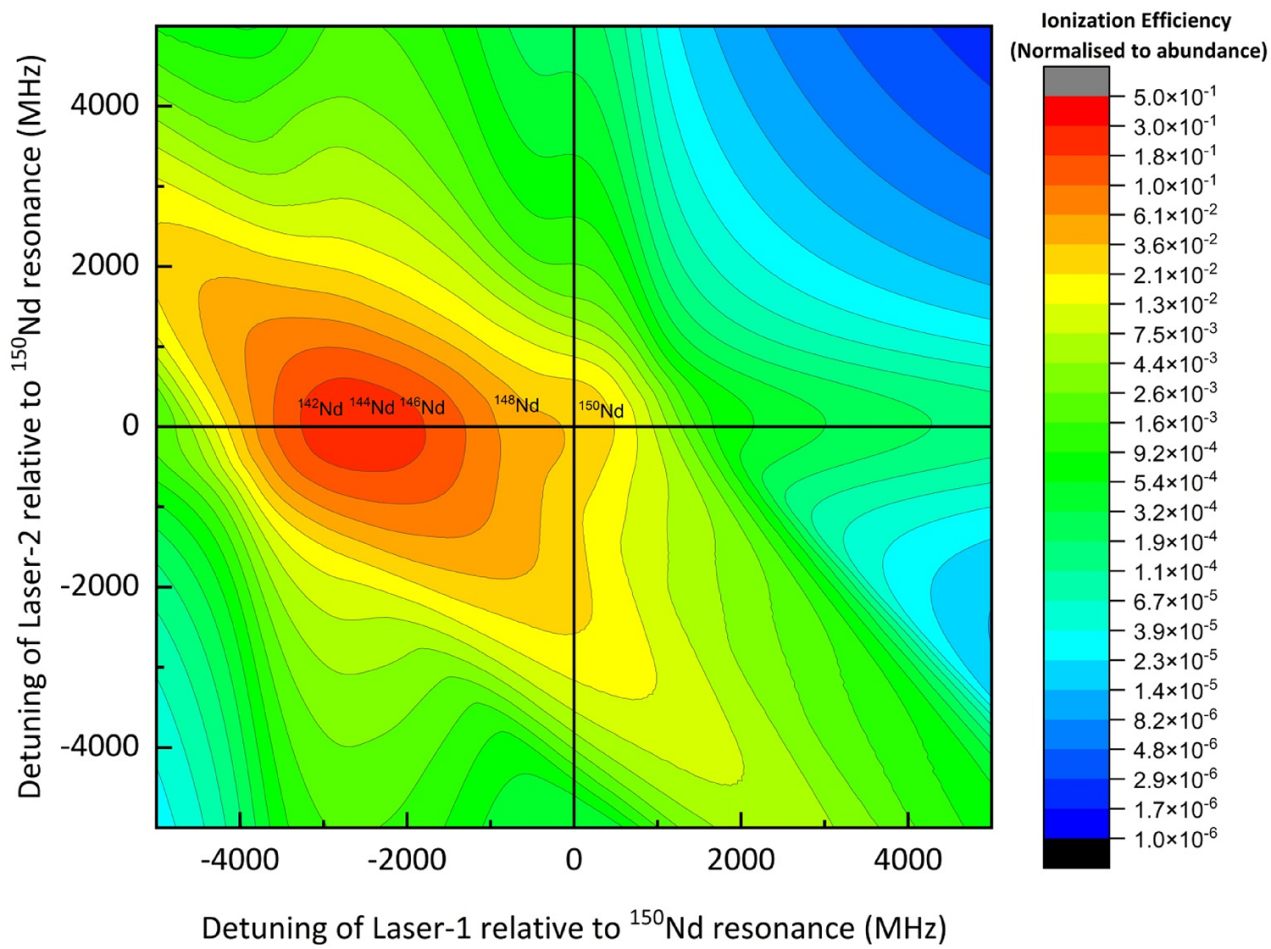
Two-dimensional contour plot of the ionization efficiencies of Nd isotopes (frequencies relative to the resonance frequency of ^150^Nd isotope). Bandwidth of the excitation lasers is set to 100 MHz, angular divergence of the atomic beam is 60° and the peak power densities of the excitation lasers are set to 20, 20 and 78,000 W/cm^2^ respectively.

A series of calculations of degree of enrichment have been carried out varying the angular divergence of the atomic beam and the bandwidth of the excitation lasers under Doppler free conditions, which have shown that the optimum peak power densities of the first, second and third lasers are 20, 20, 78,000 W/cm^2^ respectively. Under these conditions, it can be observed that the degree of enrichment is somewhat invariant with increase in the bandwidth of the excitation lasers (at least up to ≤ 150 MHz), while ionization efficiency decreases with the increase in the angular divergence of the atomic beam and the bandwidth of the excitation lasers (Table 3). This can be understood as following. At higher values of angular divergences, higher velocity groups having larger Doppler shifted resonances are less likely to be excited by the narrowband lasers. Whereas use of relatively broadband lasers somewhat compensates this loss of ionization as the higher velocity groups of the atomic ensemble can still be excited by the broadband lasers. Due to this reason, the ionization efficiency falls considerably to ~ 66% of the initial value for a laser bandwidth of 50 MHz; but just settles at ~ 36% of the initial value for a laser bandwidth of 250 MHz. At larger angular divergence values, the lineshapes are also smudged because of the Doppler broadening of the atomic ensemble (Fig. 3).

**Table 3:** A table of the degree of enrichment of ^150^Nd for various values of angular divergence of the atomic beam and for different bandwidths of the excitation lasers. Peak power densities of the first, second and third excitation lasers are 20, 20, 78,000 W/cm^2^ respectively.

Angular divergence of the atomic beam (degrees)	Bandwidth of the excitation lasers = 50 MHz		Bandwidth of the excitation lasers = 100 MHz		Bandwidth of the excitation lasers = 150 MHz		Bandwidth of the excitation lasers = 250 MHz	
	${\upeta }_{{150}_{\text{Nd}}}$	Degree of enrichment of ^150^Nd (%)	${\upeta }_{{150}_{\text{Nd}}}$	Degree of enrichment of ^150^Nd (%)	${\upeta }_{{150}_{\text{Nd}}}$	Degree of enrichment of ^150^Nd (%)	${\upeta }_{{150}_{\text{Nd}}}$	Degree of enrichment of ^150^Nd (%)
5	0.93	95.2	0.92	94.2	0.91	92.6	0.88	87.5
10	0.91	95.2	0.92	94.2	0.91	92.6	0.88	87.4
20	0.80	94.8	0.87	94.1	0.89	92.5	0.88	87.1
30	0.68	93.9	0.77	93.4	0.83	91.9	0.86	86.2
45	0.53	90.5	0.63	90.4	0.71	89.0	0.79	83.1
60	0.43	83.4	0.52	84.3	0.60	83.4	0.71	78.3
75	0.36	75.4	0.44	77.2	0.51	77.0	0.63	72.9
90	0.30	66.4	0.37	69.1	0.45	69.8	0.56	67.0

**Figure 4:**
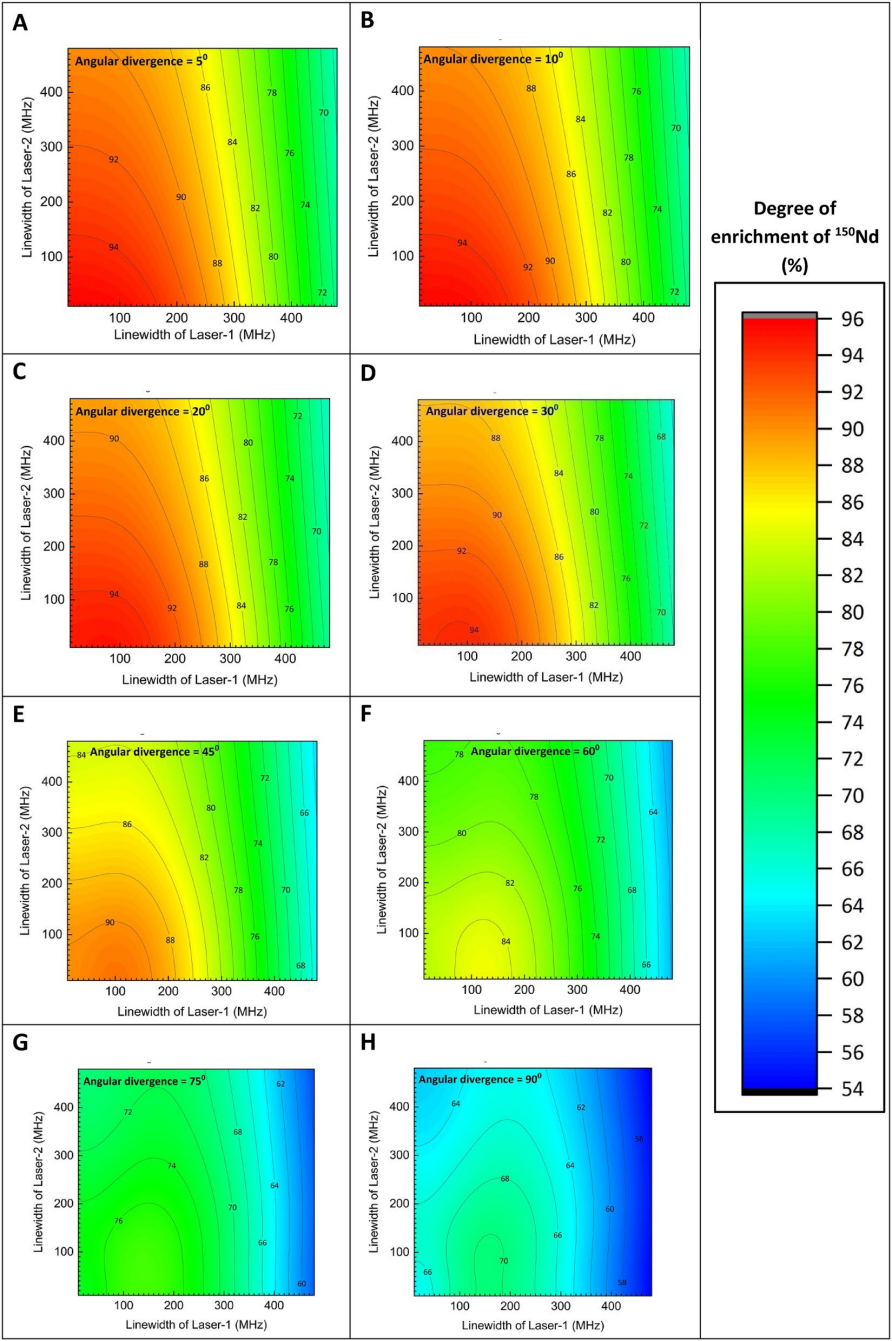
A plot of the dependence of the degree of enrichment on the bandwidth of excitation lasers calculated for various angular divergence values. Peak power densities of the first, second and third lasers have been set to 20, 20 and 78,000 W/cm^2^ respectively.

For a given bandwidth of lasers, choosing an appropriate angular divergence is important for an efficient AVLIS process. If the Doppler broadening is much smaller than the bandwidth of the excitation lasers, large portion of the laser bandwidth remains unutilized, thereby causing the loss in the photon economy of the enrichment process. On the other hand, if the Doppler broadening is much larger than the bandwidth of the excitation lasers; considerable portion of the atomic ensemble is not excited by the lasers, thereby ending up in a throughput loss of the enrichment process. On the whole, the ionization efficiency of the AVLIS process is the result of a complex interplay of the Doppler broadening, power and bandwidth of the excitation lasers. A series of computations have been carried out for various values of angular divergences of the atomic ensemble, wherein degree of enrichment is calculated by varying bandwidth of the excitation lasers (Fig. 4). When the angular divergence of the atomic beam is limited to 5° (Fig. 4A) and bandwidth of the excitation lasers is set to 100 MHz, the degree of enrichment of ^150^Nd can be as high as 94%. The high degree of enrichment can be attributed to both strict control of the angular divergence and narrow bandwidth of the excitation lasers. Here, it is important to note that further reduction in the bandwidth of the excitation lasers does not improve the degree of enrichment. On the other hand, an increase in the bandwidth of the excitation laser to 500 MHz causes a reduction in the degree of enrichment to just 70%. Similar trend has been observed up to angular divergence values of 30° (Fig. 4A–D), after which the degree of enrichment starts declining steadily. At angular divergence of 90°, the degree of enrichment varies between 66 and 55% depending on the bandwidth of the laser. Thus, it can be inferred that, angular divergence between 30° and 45° can be considered as an optimum, for obtaining a degree of enrichment between 94 and 66% using lasers with bandwidth of < 500 MHz.

### Charge exchange collisions

In atomic vapour laser isotope separation, it is important to obtain high production rates for obtaining the separation of isotopes in weighable quantities. This can be achieved only through high ionization efficiency of the photoionization process and operation of the atom source at highest number densities possible. However, the charge exchange collisions determine the limits to the operable range of the number densities. The photoions which are formed due to the laser-atom interaction in a multi-step photoionization process are extracted by applying electric field gradient. These photoions are likely to undergo charge exchange collisions with the unionized atomic vapour during the extraction. The probability of charge exchange collisions can be calculated using the equation given below^23^

19 $$\text{Probability of charge exchange }p=1-{e}^{-\sigma dN}$$

where, σ is the resonant charge exchange cross-section (cm^2^), d is the distance traversed by the photoions prior to collection at the ion collector (cm) and N is the number density of the atoms (atoms/cm^3^).

The resonant charge exchange cross-section^24^ of Nd corresponding to the atomic velocities at the temperature of 1500 °C is 1.5 × 10^–14^ cm^2^. For a number density of 1 × 10^12^ atoms/cm^3^and a 50 mm extraction length, the probability of charge exchange collisions is calculated to be 7.2%. If the number density is increased by one order i.e., to 1 × 10^13^ atoms/cm^3^, the probability of charge exchange collisions increases to 52.7% which results in a significant loss in the degree of enrichment. Therefore, it is not desirable to operate the atom source at this number density.

**Figure 5:**
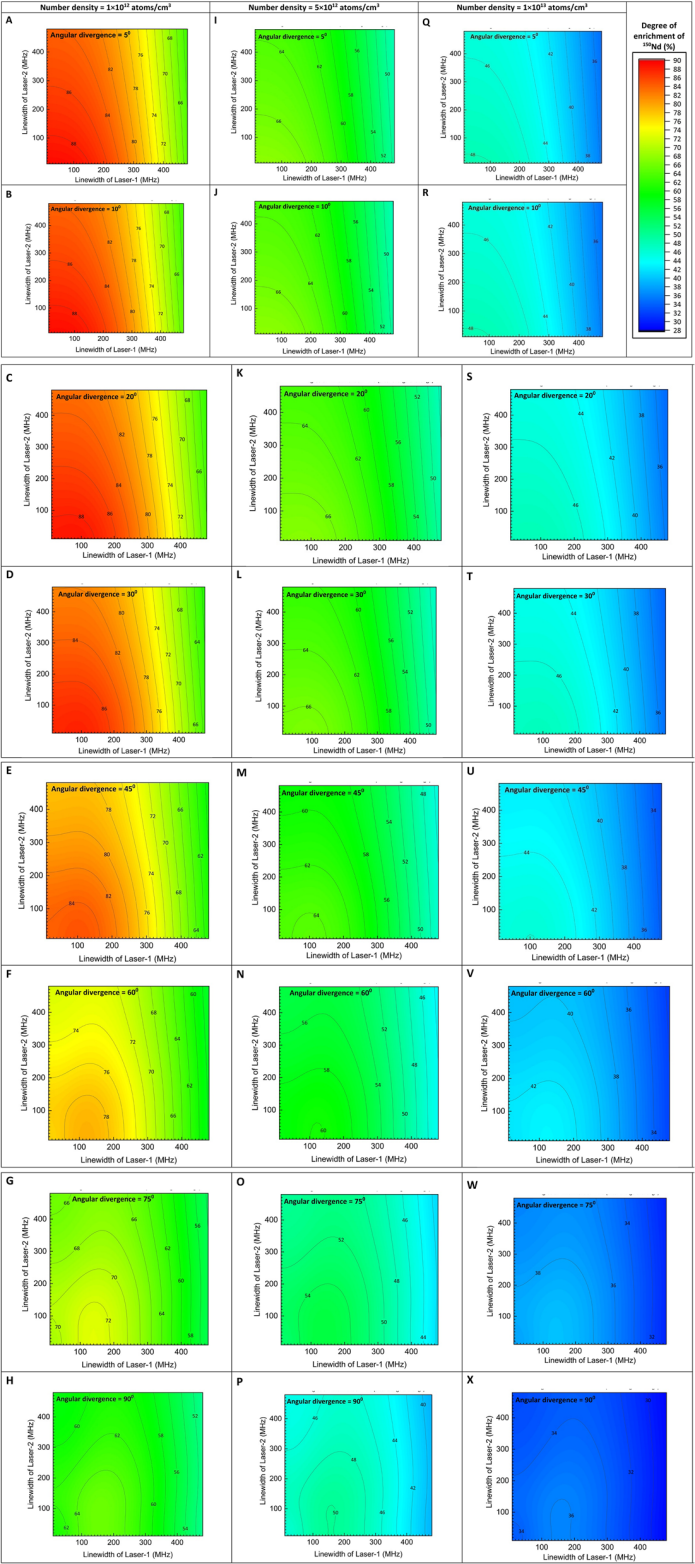
A plot of the dependence of the degree of enrichment on the bandwidth of excitation lasers calculated for different number densities. Peak power densities of the first, second and third lasers have been set to 20, 20 and 78,000 W/cm^2^ respectively.

Variation in the degree of enrichment with bandwidth of the excitation lasers has been calculated for three different number densities and the results are plotted in Fig. 5. It has been observed that, at number densities of 1 × 10^12^ atoms/cm^3^, it is possible to obtain a degree of enrichment of > 84% up to an angular divergence of 45° for a laser bandwidth of 100 MHz (Fig. 5A–E).

When the number density is 5 × 10^12^ atoms/cm^3^, the charge exchange probability increases to 31.3%. As a result, the degree of enrichment ranges between 66 and 50%, up to an angular divergence value of 30° (Fig. 5I–L). If the number density is 1 × 10^13^ atoms/cm^3^, the degree of enrichment can vary between 48 and 32% with increase in the bandwidth and angular divergence (Fig. 5Q–X).

Production rates can be calculated using the following expression

20 $$P \left(\frac{g}{hour}\right)=2 \cdot 827\times {10}^{3}\times \left({b}^{2} \cdot p \cdot l \cdot d \cdot A \cdot f \cdot \eta  \cdot i \cdot n \cdot \frac{M}{{N}_{A}} \cdot PRF\right)$$

where b is the laser beam diameter (cm), *p* fractional population of level $\left|1\rangle \right.$, l is the length of the laser-atom interaction region (cm), d is the number density of atoms in the interaction region (atoms/ cm^3^), A is the fractional abundance of the target isotope, f is the fractional flux (flux relative to the flux of unhindered atomic beam), η is the ionization efficiency (derived from the density matrix calculations), *i* is the irradiation probability, n is the number of passes of the laser beam through the atomic ensemble, M is the atomic mass of the target isotope (grams), N_A_ is the Avogadro number (6.02214076 × 10^23^) and PRF is the pulse repetition frequency of the lasers (Hz).

For the peak power densities values of the excitation lasers 20, 20 and 78,000 W/cm^2^, the ionization efficiency of ^150^Nd is 0.77 for an angular divergence of the atomic beam of 30° and for a 100 MHz bandwidth of the excitation lasers. The computed ionization efficiency was comparable to the ionization efficiency of 0.77 reported by Babichev et al.^12^.

The production rates have been calculated for the various physical parameters of the laser isotope systems and tabulated in Table 4. The Case-1 corresponds to the parameters of the isotope separation system used by Babichev et al.^12^. The calculated production rate in this case is 600 mg/h (66% enriched). The production rate can be further enhanced to ≈ 22 g/h (Case-2 of Table 4) easily by increasing the laser-atom interaction region. Currently the peak power density requirement of ionization laser is 78,000 W/cm^2^ which corresponds to an average power of 100 W for a laser beam diameter of 30 mm, pulse-width of 18 ns and a laser pulse repetition rate of 10 kHz can only be achieved by a few laboratories in the world^12,17^.

**Table 4:** A table of the production rates of enriched ^150^Nd isotope for various experimental configurations of the AVLIS systems for different laser-atom interaction lengths. Angular divergence of the atomic beam is 30°. Bandwidths of all the excitation lasers are 100 MHz.

Description	Case-1	Case-2
Laser beam diameter (mm)	30	30
Laser-atom interaction region length (mm)	270	10,000
Number density (/cm^3^)	5 × 10^12^	5 × 10^12^
Ionization efficiency	0.77	0.77
Irradiation probability	0.15	0.15
PRF (Hz)	10,000	10,000
Number of passes	20	20
Degree of enrichment	66%	66%
Production rate (g/h)	0.6	22
Number of working hours required for production of 50 kg of enriched isotope	8.3 × 10^4^	2.3 × 10^3^

## Conclusions

A 562 nm–627 nm–597 nm three-step resonant photoionization scheme has been studied for the enrichment of ^150^Nd in weighable quantities for the neutrinoless double beta decay detection. The optimum conditions for the enrichment process have been derived using the density matrix formalism. It has been shown that it might be possible to produce 50 kg of 66% enriched ^150^Nd isotope in about five months (16 h/day) using the conditions derived through this investigation. Peak power density requirement of the ionization laser is 78,000 W/cm^2^ which corresponds to an average power of 100 W for a laser beam diameter of 30 mm, pulse-width of 18 ns and a laser pulse repetition rate of 10 kHz laser can only be achieved by a few laboratories in the world. The effect of bandwidth of the excitation laser and charge exchange collisions, on the production rates and degree of enrichment have been studied. It is also been observed that the spectroscopic data of Nd such as data on autoionization levels and cross-sections, lifetimes, branching ratios, isotopes shifts of upper level transitions have still not been adequately studied or reported. Perhaps further work in this area is required to enable researchers to find more efficient photoionization pathways which require less demanding conditions for the laser isotope separation of Nd in large quantities.

## Data Availability

Data underlying the results presented in this paper are not publicly available at this time but may be obtained from the authors upon reasonable request.

## Code availability

No commercial software has been used for the computations. The source code developed by the author has been used for the computations.
